# Optical Coherence Tomography in Alzheimer’s Disease and Other Neurodegenerative Diseases

**DOI:** 10.3389/fneur.2017.00701

**Published:** 2017-12-19

**Authors:** Jonah Doustar, Tania Torbati, Keith L. Black, Yosef Koronyo, Maya Koronyo-Hamaoui

**Affiliations:** ^1^Department of Neurosurgery, Maxine Dunitz Neurosurgical Research Institute, Cedars-Sinai Medical Center, Los Angeles, CA, United States; ^2^College of Osteopathic Medicine of the Pacific, Western University of Health Sciences, Pomona, CA, United States; ^3^Department of Biomedical Sciences, Cedars-Sinai Medical Center, Los Angeles, CA, United States

**Keywords:** spectral domain, optical coherence tomography, beta-amyloid, alpha-synuclein, retinal imaging, Parkinson’s disease, multiple sclerosis, Huntington’s disease

## Abstract

Over the past decade, a surge of evidence has documented various pathological processes in the retina of patients suffering from mild cognitive impairment, Alzheimer’s disease (AD), Parkinson’s disease (PD), and other neurodegenerative diseases. Numerous studies have shown that the retina, a central nervous system tissue formed as a developmental outgrowth of the brain, is profoundly affected by AD. Harboring the earliest detectable disease-specific signs, amyloid β-protein (Aβ) plaques, the retina of AD patients undergoes substantial ganglion cell degeneration, thinning of the retinal nerve fiber layer, and loss of axonal projections in the optic nerve, among other abnormalities. More recent investigations described Aβ plaques in the retina located within sites of neuronal degeneration and occurring in clusters in the mid- and far-periphery of the superior and inferior quadrants, regions that had been previously overlooked. Diverse structural and/or disease-specific changes were also identified in the retina of PD, Huntington’s disease, and multiple sclerosis patients. The pathological relationship between the retina and brain prompted the development of imaging tools designed to noninvasively detect and monitor these signs in living patients. One such tool is optical coherence tomography (OCT), uniquely providing high-resolution two-dimensional cross-sectional imaging and three-dimensional volumetric measurements. As such, OCT emerged as a prominent approach for assessing retinal abnormalities *in vivo*, and indeed provided multiple parameters that allowed for the distinction between normal aged individuals and patients with neurodegenerative diseases. Beyond the use of retinal optical fundus imaging, which recently allowed for the detection and quantification of amyloid plaques in living AD patients *via* a wide-field view of the peripheral retina, a major advantage of OCT has been the ability to measure the volumetric changes in specified retinal layers. OCT has proven to be particularly useful in analyzing retinal structural abnormalities consistent with disease pathogenesis. In this review, we provide a summary of OCT findings in the retina of patients with AD and other neurodegenerative diseases. Future studies should explore the combination of imaging early hallmark signs together with structural–functional biomarkers in the accessible retina as a practical means of assessing risk, disease progression, and therapeutic efficacy in these patients.

## Introduction

Shielded against external injuries, the brain is a concealed central nervous system (CNS) structure that may be uniquely observed through the one exception to its enclosure: the retina. The neuro retina, a developmental outgrowth of the embryonic brain, contains multiple molecular and cellular features in common with the brain, including neurons, glial cells, connected vasculature, and the existence of a blood barrier (1–4). The tight link between these two CNS tissues raises the prospect of whether the easily accessible retina may faithfully represent the brain in healthy and neurodegenerative conditions.

As the most common form of neurodegeneration, Alzheimer’s disease (AD) is an invariably fatal senile dementia with no cure and limited ability for early unequivocal diagnosis in primary care settings (5). AD is clinically represented by severe cognitive decline, socio-behavioral manifestations, and various visual dysfunctions involving narrowed visual field, reduced contrast and color recognition, and circadian sleep-wake disturbances (6–10). Its neuropathology is a complex continuum of detrimental processes, most likely beginning with the accumulation and propagation of misfolded amyloid β-protein (Aβ) assemblies followed by the hyperphosphorylation of (p)tau proteins forming neurofibrillary tangles (NFTs) (11). These processes are thought to initiate a cascade of secondary pathologies including destructive inflammatory responses, vascular-associated abnormalities, oxidative stress, mitochondrial dysregulation, and vast synaptic and neuronal loss (12–14). While these secondary indicators are shared among several neurodegenerative diseases, they may indicate disease progression, denoting functional deterioration and clinical staging. Importantly, growing evidence indicates that AD is not confined to the brain but also massively affects the retina, an organ feasible for direct, high-resolution imaging [reviewed in Ref. (15)].

Over the past three decades, corresponding pathologies associated with tissue degeneration were found in the retina of patients with neurodegenerative diseases [reviewed in Ref. (2, 15, 16)]. In particular, severe optic nerve and retinal ganglion cell (RGC) degeneration, thinning of the retinal nerve fiber layer (RNFL), and abnormal electroretinography responses were documented in patients with mild cognitive impairment (MCI) and AD, perhaps offering an effective measure of neurodegenerative progression. Similar to AD, other neurological diseases have been shown to affect the retina in certain geometric locations and cellular layers. This pathological relationship between the retina and brain prompted the development of imaging tools designed to noninvasively detect and monitor these signs in living patients.

One such tool, optical coherence tomography (OCT), is a unique technology providing high-resolution (1–15 µm) two-dimensional cross-sectional imaging and three-dimensional volumetric measurements. As such, OCT is emerging as a prominent technique for assessing retinal abnormalities *in vivo*, utilizing the reflective and optical properties of tissue against a long wavelength light beam for analysis and distinction of layers (17–22). Several generations of enhancements have produced different modalities such as Fourier/frequency domain OCT (fd-OCT), stratus-OCT, and spectral domain OCT (sd-OCT), which has distinct advantages in resolution and signal-to-noise ratios above time domain OCT (19, 23–25). In recent years, multiple studies have acknowledged the benefits of these advancements, particularly in AD, Parkinson’s disease (PD) and Huntington’s disease (HD). Along with future technological improvements, the ability of OCT to provide detailed data on retinal atrophy may prove to be a useful technique for assessing neurodegenerative attributes in patients, both post- and presymptomatic.

While the pathological hallmarks of AD—Aβ plaques and NFTs—have been well established in the brain for over a century, their existence in the retina has only recently been identified (26–28). Likewise, accumulation of the PD pathological hallmark α-synuclein was newly shown in retinal tissues of patients (29). Disease-specific protein aggregates are definitive signs that could facilitate differential diagnosis between various neurodegenerative diseases affecting the CNS. In particular, cerebral Aβ deposits are asserted as the earliest pathognomonic risk factor for AD, evidenced by studies detecting accumulation as early as 20 years prior to the onset of clinical dementia, an insidious phase denoted as prodromal AD (12, 30, 31). Recent identification of retinal Aβ deposits in concurrence with RGC degeneration in AD patients (26, 28) combined with the accessibility of the retina for noninvasive high-resolution imaging offers hope for prodromal phase intervention and effective treatment. This review covers key pathological findings in the retina of AD and other neurodegenerative diseases, as well as current investigational retinal imaging approaches to detect disease in living patients, with a focus on the utility of OCT.

## Retinal Pathology in AD

### Evidence of Retinal Degeneration in AD

Growing evidence suggests that various cerebral pathologies associated with AD also exist in the retina of MCI and AD patients (15, 21, 32–51). In 1986, Hinton et al. reported findings of optic nerve degeneration in 8/10 AD patients, of which 3/4 patient retinas also displayed ganglion cell layer (GCL) loss and thinning of the RNFL (52). In a follow-up study, Blanks et al. (53) confirmed the original report in 14/16 AD patients, with a significant loss of RGCs and their axons as well as vacuolar degeneration occurring within these cells (53). An investigation by Sadun and Bassi (54) suggested similar findings, while highlighting a predominant loss of M-cell class RGCs (54). A later study showed an extensive decrease in ganglion cell number in the foveal and parafoveal retina (55). Examination of flatmount retinas including peripheral regions from 11 AD patients and 9 controls revealed a substantial >36% overall neuronal loss throughout the retina, which was more pronounced in the superior and inferior quadrants, specifically in the mid-peripheral regions (40–49%) and far-peripheral inferior retina (50–59%) (56).

With regard to inflammation and vascular changes, retinal astrogliosis was described alongside neuronal loss in the retina of AD patients (56). Subsequent examination of AD retina added support for structural changes in inner retinal layers (41, 57), and further uncovered a host of alterations in cup-to-disk ratio, macular volume, blood flow, and vasculature (15, 58–64). Of great interest were numerous findings of retinal angiopathy and vascular-related changes similar to cerebral AD pathologies, including narrowed veins, reduction of blood flow, elevated blood oxygen saturation, and increased tortuosity (36, 59, 60, 64–66). Converging data on AD-related retinal abnormalities encouraged investigators to develop noninvasive retinal imaging modalities in order to detect and measure these anatomical changes in living patients. This was initially attempted using blue-light high-resolution photography by Tsai et al. (63), and later advanced to utilizing cross-sectional imaging by OCT (34, 37, 67–70). OCT findings in MCI, AD, and PD patients along with their correlations with cognition and vision are described subsequently and summarized in Tables 1 and 2.

**Table 1:** OCT findings in the retina of AD, MCI, and PD patients.

Clinical diagnosis	Region	OCT type	% reduction range (min − max Δ values)^a^	Reference
Findings				
**Alzheimer’s disease**				
RNFL thinning	All quadrants	sd-OCT, fd-OCT, stratus-OCT	6.8–40.4% (6.61–40.40 µm)	(28, 37, 38, 41–43, 67, 70–73),* (44, 74–77),** (78–80)^nd^
	Superior		7.7–51.9% (6.04–118.20 µm)	(28, 33, 38, 41, 42, 65, 67, 70, 72–74, 81),* (77),** (82),*** (75, 79)^nd^
	Inferior		9.9–33.0% (5.40–38.30 µm)	(38, 41, 42, 67, 72, 73, 81),* (74),** (82),*** (75, 79)^nd^
	Temporal	sd-OCT	10.0–55.7% (5.10–47.70 µm)	(38, 67, 81),* (78),** (75, 79)^nd^
	Nasal		8.0–46.0% (5.75–43.00 µm)	(67, 72, 81),* (75, 79)^nd^
Retinal thinning (inner and outer sectors)	Macula/fovea	sd-OCT, fd-OCT	5.7–13.4% (9.23–25.24 µm)	(38, 70, 71, 78, 79, 83),* (74, 82)**
Reduced volume			2.7–3.4% (0.20–0.34 mm^3^)	(38, 83)*
GCL-IPL thinning	All quadrants	sd-OCT, fd-OCT	8.3–8.7% (4.21–8.60 µm)	(33, 71),* (37, 74)**
Amyloid deposits; inclusion bodies; autofluorescent spots	Superior/inferior Periphery	sd-OCT	Deposit/lesion detection	(26, 48, 84)
**Mild cognitive impairment**				
RNFL thinning	All quadrants	sd-OCT, stratus-OCT	4.7–12.6% (4.75–12.90 µm)	(37, 41–43),* (44)**
	Superior	stratus-OCT	3.3–8.3% (3.96–10.13 µm)	(38, 42)*
	Inferior		11.9% (15.10 µm)	(41)**
	Temporal	sd-OCT	10.8% (8.04 µm)	(38)*
Reduced volume	Macula/fovea	sd-OCT	3.3% (0.33 mm^3^)	(38)*
GCL-IPL thinning	All quadrants	sd-OCT	nd (3.62–5.83 µm)	(33, 37)*
**Parkinson’s disease**				
RNFL thinning	All quadrants	sd-OCT, stratus-OCT	13.8% (15.78 µm)	(85),* (76),** (45)^ns^
	Superior	sd-OCT, fd-OCT, stratus-OCT	2.3–9.6% (3.05–13.30 µm)	(19),* (86)**
	Inferior	fd-OCT	6.2–15.0% (8.40–26.00 µm)	(19, 87),* (88)**
	Temporal	sd-OCT	7.1–19.8% (4.98–25.00 µm)	(88),* (86)**
	Inferotemporal		5.1–5.5% (7.02–7.88 µm)	(86)**
	Nasal	stratus-OCT	23.6% (23.51 µm)	(19)*
Retinal thinning	Macula/fovea	sd-OCT, fd-OCT	2.8–4.0% (7.50–10.80 µm)	(19, 85, 89),* (86)**
Reduced volume		stratus-OCT	3.7% (0.27 mm^3^)	(85)*
Inner retinal layer	All quadrants	sd-OCT, fd-OCT	13.9% (14.44 µm)	(90, 91)*

*^a^Reduction in percent and tissue volume/thickness of mean values between patients and healthy controls (range values)*.

*p < 0.05.

**p < 0.001.

***p < 0.0001.

*nd, statistical data not shown; ns, a trend, not statistically significant; NA, not applicable; sd-OCT, spectral domain-OCT; fd-OCT, frequency domain-OCT*.

**Table 2:** Correlations between OCT findings and clinical dysfunction/progression in AD and PD patients.

Clinical diagnosis	Region	OCT type	Degree of correlation^a^	Reference
Correlations				
**Alzheimer’s disease/mild cognitive impairment**				
RNFL thickness vs. cognitive function^b^	All quadrants	sd-OCT, fd-OCT	*r* = 0.33	(43)*, (74, 76)**
	Superior	fd-OCT	*r* = 0.24	(74)*
	Inferior	sd-OCT, fd-OCT	*r* = 0.35–0.65	(50)*, (74)**
	Temporal	NA	NA	NA
	Nasal			
GCL-IPL thickness vs. cognitive function^b^	All quadrants	fd-OCT	*r* = 0.33–0.49	(37, 74)*
Macular thickness vs. cognitive function^b^	All quadrants	sd-OCT, fd-OCT	*r* = 0.34	(74)*, (79)**, (38, 41, 75, 92)^ns^
	Superior	fd-OCT	*r* = 0.47	(74)**
	Inferior		*r* = 0.46	
	Temporal		*r* = 0.49	
	Nasal		*r* = 0.48	
RNFL thickness vs. visual function	All quadrants	sd-OCT	*r* = 0.46–0.76	(67, 70, 93, 94)*, (79)^ns^
**Parkinson’s disease**				
RNFL thickness vs. disease progression	All quadrants	sd-OCT, stratus-OCT	*r* = 0.39–0.66	(85, 86)*
RNFL thickness vs. visual function		sd-OCT	*r* = 0.40	(86)**, (87)***

*^a^Pearson (*r*) correlations are represented as absolute values*.

*^b^Cognitive function examinations: ADAS-cog, CDR, and MMSE*.

*p < 0.05.

**p < 0.001.

***p < 0.0001.

*nd, statistical data not shown; ns, a trend, not statistically significant; NA, not applicable; sd-OCT, spectral domain-OCT; fd-OCT, frequency domain-OCT; ADAS-cog, AD Assessment Scale-cognition; CDR, Clinical Dementia Rating; MMSE, Mini-Mental State Examination*.

### AD Hallmark Pathology in the Retina

While structural and vascular alterations observed in the retina of AD patients may prove essential for predicting functional decline or conversion to symptomatic AD, they could be shared among several neurodegenerative diseases. It was not until recently that the existence of AD-specific hallmarks, Aβ plaques, was revealed by Koronyo-Hamaoui et al. (27) in 13/13 postmortem retinas of definite AD patients and early-stage cases, in a stark contrast to 5 healthy controls (27). Corroborating these findings, several other groups later demonstrated elevated Aβ peptides, forms of Aβ deposits, and the presence of hyperphosphorylated (p)tau in retinas of AD patients but not in controls (26, 28, 95–98). Scanning of large flatmount retinal sections, especially of mid- and far-peripheral regions, allowed for the identification of diverse retinal Aβ plaque morphologies that were often associated with blood vessels or colocalized with sites of cell degeneration (26–28). Retinal plaque pathology mirrored amyloid pathology in the brain, including the vascular amyloid component and the existence of classical and neuritic plaques as well as proto-fibrils and fibrils comprised of Aβ_42_ alloforms (26, 27).

**Figure 1:**
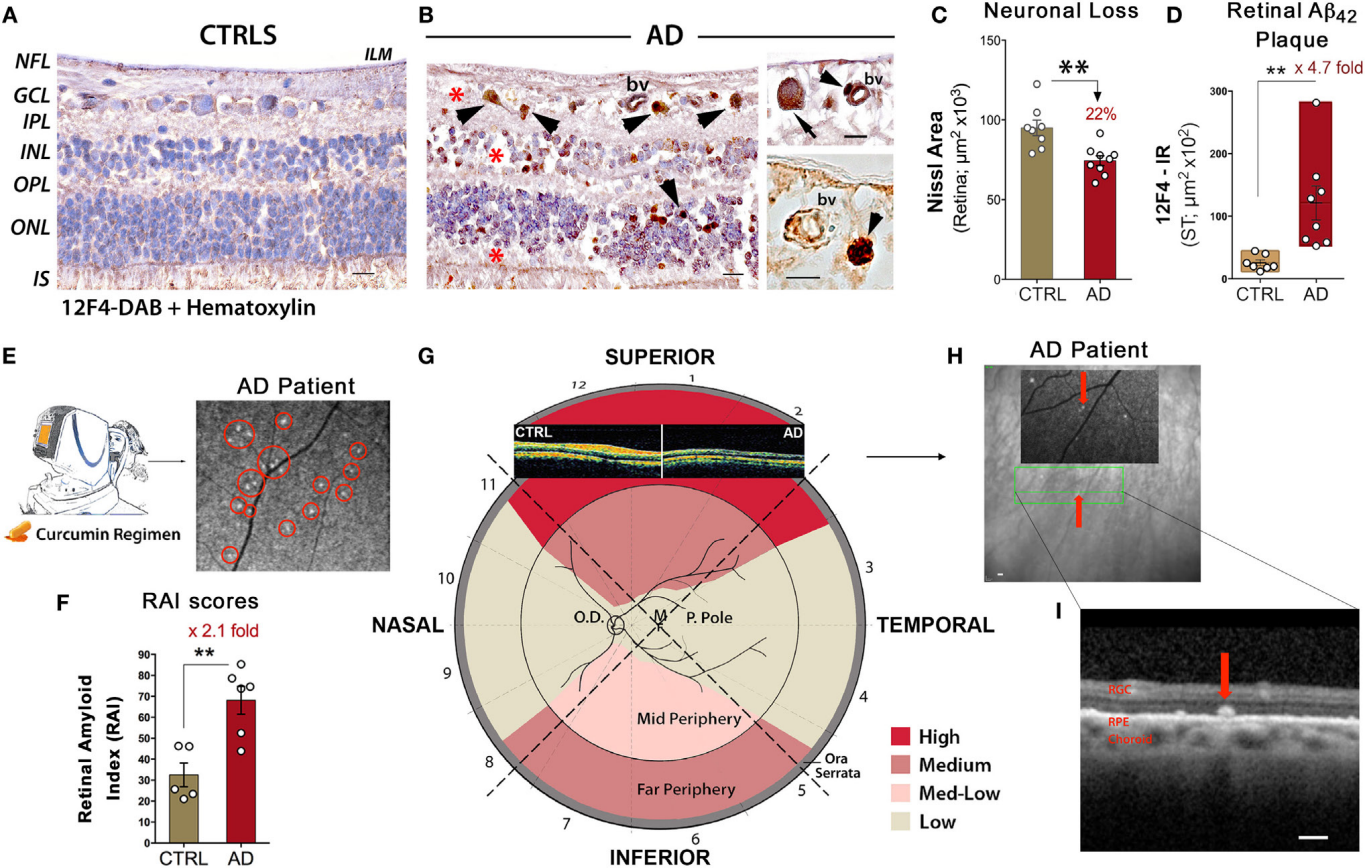
Noninvasive retinal imaging in Alzheimer’s patients: detecting Aβ deposits and nerve degeneration *via* scanning laser ophthalmoscopy and optical coherence tomography. **(A,B)** Retinal cross sections from superior quadrants of Alzheimer’s disease (AD) patients (*n* = 12) and matched healthy controls (CTRL; *n* = 8) stained with anti-Aβ_42_ mAbs (12F4) and peroxidase-based labeling (brown). Hematoxylin counterstain for nuclei (violet). Retinas of AD patients contained a multitude of Aβ deposits (arrowheads), especially in the ganglion cell layer (GCL). Marked loss of retinal cells is apparent in the GCL, inner nuclear layer (INL), and outer nuclear layer (ONL); areas of nuclei loss are indicated by red asterisks. The inner limiting membrane (ILM) and retinal nerve fiber layer (RNFL) are intact in CTRL in contrast to AD. Scale bars = 20 μm. Higher magnification images [**(B)**, right panel] show Aβ deposits near and inside blood vessel walls (bv; arrowheads) and inside ganglion cell soma (arrow). Scale bars = 10 μm. **(C)** Quantitative Nissl neuronal area in AD patients (*n* = 9) and age- and gender-matched CTRL (*n* = 8) revealing a significant reduction in AD patients. **(D)** Quantitative analysis of 12F4-immunoreactive (IR) area of Aβ_42_-containing plaques in the superior quadrant of retinal flatmounts in a subset of definite AD patients (*n* = 8) and matched controls (*n* = 7) showing a significant increase of Aβ_42_ plaques in AD patients. **(E)** Schematic of a noninvasive retinal amyloid imaging method using curcumin (Longvida^®^) to label retinal Aβ in live human patients. Subjects’ retinas were imaged with a modified scanning-laser ophthalmoscope prior to and following curcumin intake. White spots marked by red circles are curcumin-positive amyloid plaques detected in the retina of a living AD patient. **(F)** Scatter bar plot displays retinal amyloid index (RAI) scores, a fully automated calculation of increased curcumin fluorescence representing amyloid deposits in the retina. AD patients (*n* = 6) showed a significant increase in RAI score in comparison to age-matched CTRL (*n* = 5). **(G)** Qualitative mapping of the “geometric hotspot” regions of Aβ deposits in retinal quadrants of AD patients. Schematic of OCT images from an AD patient vs. control are shown [adopted from: Coppola et al. (21)]. **(H)** OCT image of a selected curcumin-positive plaque (red arrow) in an AD patient with no maculopathy. Certain retinal amyloid plaque visualized by curcumin fluorescence fundography in insert. Green lines delineate region of OCT segmentation. **(I)** Retinal cross section by OCT reveals amyloid plaque in outer retinal layers; curcumin-positive deposit located above retinal pigment epithelium (RPE), along with intact RPE and Bruch’s membrane. Scale bars = 200 μm. Group means and SEMs are shown. *p < 0.05 and **p < 0.01, unpaired two-tailed Student’s *t*-test. Images and data of **(A–I)** panels, except for part of **(E)**, are modified reprinted from Koronyo et al. (26).

Cross sections isolated from regions rich in Aβ pathology showed that these plaques occur in patients more abundantly in inner retinal layers, especially in the GCL, while colocalizing around and within degenerating RGCs (see Figures 1A–D) (26). Curcumin-based fundus imaging in 10 living AD patients versus 6 healthy controls showed that, in agreement with histologic examination of additional retinas from 23 definite AD patients versus 14 healthy controls, retinal Aβ deposits appear in clusters and are frequently mapped to peripheral regions of the superior and inferior quadrants (see Figures 1E–G) (26). These regions were often overlooked due to their perceived irrelevance in common retinopathies [i.e., age-dependent macular degeneration (AMD), glaucoma] and visual acuity (VA).

A quantitative histological analysis of Aβ_42_-containing retinal plaques in 8 confirmed AD patients and 7 age- and gender-matched controls indicated a substantial 4.7-fold increase of plaque burden in the retina of patients (Figure 1D), correlating with Aβ burden in the respective brains (26). Similarly, noninvasive curcumin fundus imaging in living patients showed a 2.1-fold increase in retinal amyloid index (RAI) scores in a subset of AD patients versus matched controls (Figure 1F) (26). The discovery of classical and neuritic-like plaques, albeit smaller in size compared to plaques in the brain, along with NFTs, Aβ_42_ fibrils, protofibrils, and structures resembling oligomers, suggests that the specific signs of AD are shared between the retina and the brain (26). Importantly, in a clinical study, circadian dysfunctions were found in AD patients along with structural OCT changes, especially in the superior quadrant (28). Degeneration of (melanopsin-containing) mRGCs, photoreceptors known to drive circadian photoentrainment, was further shown to be associated with retinal Aβ deposits in AD patients [reviewed in Ref. (99)].

In agreement with findings in patients, retinal Aβ deposits were identified in various transgenic and sporadic rodent models of AD at different disease stages [reviewed in Ref. (15, 100)] (27, 98, 101–107). Retinal Aβ was associated with RGC degeneration, local inflammation, and functional impairments. Transgenic mouse models of AD subjected to immunotherapy (108–111) exhibited reduction of Aβ plaque burden in the retina to the same extent as in the brain (27, 104, 105, 112). To visualize *in vivo* retinal Aβ pathology in animal models and AD patients, our group developed a noninvasive retinal amyloid imaging method, using curcumin as a fluorescent probe binding to Aβ deposits (26, 27, 104). This approach enabled noninvasive, longitudinal monitoring of individual Aβ deposits, their appearance during disease progression, and their clearance following immunomodulation therapy in mice (104). The evidence of atrophic and proteinaceous pathology in animal models and AD patient retinae, described above, provides the rationale for the application of OCT retinal imaging in neurodegeneration detection.

## OCT Findings in AD

### RNFL Thinning

Current OCT research investigating visual system degeneration in various neurological diseases produces compelling evidence for retinal thinning, particularly in MCI and AD (see summary in Table 1). While a subset of these studies have shown specific deterioration of the GCL along with the inner plexiform layer (IPL) (33, 37, 71, 74), the majority have demonstrated thinning of the RNFL, especially in the superior and inferior quadrants, with a focus on the peripapillary region [peripapillary retinal nerve fiber layer (pRNFL)] surrounding the optic disk (21, 34, 35, 37, 50, 65, 68, 71, 74, 78, 83, 92, 113, 114). As the inner-most layer of the ocular fundus, this area is composed of RGC axonal projections leading to the optic nerve. Parisi et al. (67) were the first to describe retinal abnormalities in AD patients by utilizing cross-sectional imaging by OCT (67). With modern advancements in noninvasive imaging technologies including next-generation OCT methods along with the identification of pathological hallmarks in the retina, there has been an exponential growth of investigations into retinal pathology in AD patients, especially using OCT. A recent report extensively assessed quadrant-specific circumpapillary RNFL and macular thickness as well as macular volume in AD patients compared to healthy controls (AD, *n* = 18; HC, *n* = 41) (32). Significant thinning in all quadrants—superior, inferior, nasal, and temporal—of the circumpapillary RNFL (*p* < 0.001) of both left and right eyes was detected, in addition to inner and outer macular ring thinning.

Although a couple of studies failed to find differences in retinal thickness between patients with AD or MCI and matched HC (45, 92), many more studies detected significant RNFL structure abnormalities in AD and MCI patients compared with matched HC (32, 37, 38, 41–44, 46, 50, 67, 71–73, 75, 76, 78, 79, 81, 82, 87, 114–116). These reports have indicated that RNFL thickness in the patients was substantially decreased in all quadrants or that there were specific reductions in the supraretina and infra-retina. Moreover, meta-analysis studies support the important role of OCT for RNFL analysis in monitoring the progression of AD by reporting that retinal thickness was significantly decreased in AD and MCI patients compared to HC (21, 35). Results of OCT studies in MCI and AD patients are summarized in Table 1.

Although measurements of RNFL thinning in AD demonstrates promise as a means of assessing degeneration, investigation of distinct AD effects in certain retinal regions could enhance the practicality of OCT in diagnosis, since some overlap was reported between OCT findings in AD and other neurodegenerative diseases. One study reported a significant decrease in RNLF thickness in AD, dementia with Lewy bodies, and dementia associated with PD, compared to HC group; however, there was no significant difference between types of dementia (76). In addition, patients with frontotemporal dementia exhibited a significant pRNFL reduction, similar to AD and MCI patients (37). In a more recent multisite study, 21 AD patients were compared against 74 age-matched controls, revealing a significant (*p* = 0.038) reduction in average RNFL thickness *via* OCT imaging. A detailed investigation into region-specific degeneration showed that the superior quadrant presented the most significant reduction [*p* = 0.006 (28)]. A continued theme of quadrant-specific RNFL thinning in the superior region has been reported in multiple AD retinal studies (33, 41, 42, 65, 68, 70, 73, 77). Marked RNFL thinning in the superior quadrant in AD patients may significantly correlate with disease stage and support the important role of OCT for RNFL analysis in monitoring AD progression.

### Correlation of RNFL Thickness with Visual-Related Dysfunction in AD

To explore a possible relationship between OCT-detected retinal structural abnormalities and visual dysfunctions in AD patients, a correlation between RNFL thinning and retinal electrical response *via* pattern electroretinogram (PERG) was investigated in these patients (67). Specifically, each participant was exposed to a controlled, standardized stimulus, with amplitudinal voltage response as the measured dependent variable. Response time of N35, P50, and N95 were significantly delayed with the amplitudinal difference from N35-P50 and P50-N95 also equally impaired in AD subjects (67). When these data were compared against RNFL thickness, significant correlations between overall RNFL and PERG P50 and N95 implicit times as well as P50-N95 amplitude were revealed. A follow-up study from the same research group found similar correlations between retinal structure and function among patients affected by ocular hypertension glaucoma, demyelinating optic neuritis (MSON), and AD (93). Those patients who exhibited abnormal PERG responses with delayed implicit times and reduced amplitudes also had a significant reduction in NFL thickness compared to controls (93).

A later study by Iseri et al. (79) did not find a significant difference in the latency of visual-evoked potential P100 between AD patients and control subjects, with no correlation to RNFL thinning (79). Of note, Moschos et al. (82) reported a decrease in macular and RNFL thickness along with reduced multifocal-ERG activity of the macula in patients with AD, even in those without visual deficits (82).

Impairments in contrast-sensitive vision (CSV), VA, and color vision (CV) in AD patients were demonstrated in a study by Polo et al. (70), together with a strong correlation to sd-OCT measurements (70). In addition to superior quadrant thinning of the RNFL, both CSV and CV significantly worsened in AD patients as compared to controls. CSV was the functional parameter most strongly correlated with structural measurements in patients with AD; CV was strongly associated with macular volume. VA at different levels of contrast was associated with macular and RNFL thickness in AD (70). The correlations between OCT findings and visual-related changes are summarized in Table 2. Future examination of morphological and pathological origins of ocular functional decline should be performed to establish the association between visual-related dysfunction and RNFL thickness in AD.

### Correlation of Retinal Thickness and Cognitive Function in MCI and AD

An intriguing study by Ascaso et al. (32) described that the retinal thinning observed in MCI patients was further exacerbated in AD patients. A correlation between RNFL thickness and cognitive assessments such as the Mini-Mental State Examination (MMSE), Alzheimer’s Disease Assessment Scale-cognition (ADAS-cog), or Clinical Dementia Rating (CDR) could help to conclusively determine the coprogression of RNFL thinning and cognitive decline (32). Indeed, in a new study by Ferrari et al. (37) in which AD and MCI patients exhibited a significant reduction in RNFL and GCL-IPL compared to HC, the GCL-IPL thickness measurement correlated with MMSE scores without significant effects of age, gender, or disease duration. Correlation with disease severity in AD suggested that retinal and brain neurodegeneration may occur in parallel to some extent (37). Furthermore, Cunha et al. (74) revealed that there is a significant correlation in AD patients between MMSE scores and pRNFL thickness in average (*p* = 0.001), superior quadrant (*p* = 0.019), and inferior quadrant thickness (*p* < 0.001) (74). Significant correlations were also noted in various full-length macular measurements (i.e., average thickness, *p* = 0.001) as well as GCL-IPL layer thickness (*p* = 0.001) (74).

The relationship between the degree of cognitive impairment and RNFL thickness was further studied by Oktem et al. (43). RNFL thickness was significantly lower in AD and MCI groups compared with the HC group; a significant correlation was found between MMSE scores and RNFL thickness values (43). To investigate the potential association between RNFL thickness and episodic memory in MCI patients, Shen et al. (46) reported that in MCI patients, inferior quadrant RNFL thickness was inversely associated with the following episodic memory scores: word list learning (*r* = −0.652, *p* = 0.001), story memory (*r* = −0.429, *p* = 0.041), and story recall (*r* = −0.502, *p* = 0.015) (46). A summary of correlation analyses between OCT measurements and cognitive dysfunctions is displayed in Table 2.

Interestingly, Iseri et al. (79) found that reduction in macular volume in AD patients was linked to severity of cognitive impairment; total macular volume and MMSE scores were significantly correlated (79). A longitudinal case study spanning 12 months investigated whether OCT measurements of AD patients would correlate with progressive changes in cognition as determined by multiple cognitive exams (50). In particular, significant correlations between the change in pRNFL thickness and the shift in ADAS-cog (*r* = −0.35, *p* = 0.02) and CDR scores (*r* = −0.39, *p* = 0.008) were found (50). Since a few studies resulted in no significant correlation between cognition and OCT-derived degeneration (38, 41, 75, 92), further careful investigation is needed to determine whether OCT can be used to determine neurodegeneration as the source of cognitive dysfunction in AD.

### Noninvasive Retinal Amyloid Imaging in AD

Optical coherence tomography is a valuable and useful *in vivo* technique for determining retinal structural abnormalities and cell neurodegeneration in AD patients, and may help to distinguish between HC, AD patients, and other neurodegenerative diseases by the measure of RNFL thinning, specifically in the superior and inferior quadrants. Yet, definitive diagnosis of AD requires the hallmark specific identification of Aβ and NFTs. In recent years, a few studies have been able to detect retinal amyloid deposits, inclusion bodies, and autofluorescent spots in live subjects *via* a modified scanning laser ophthalmoscope (SLO), fundus autofluorescence (FAF) imaging, and/or OCT. In a clinical study, Kayabasi et al. (84) examined 30 MCI patients with FAF and OCT and claimed to detect abnormal Aβ deposits, mostly in the outer plexiform, ganglion, and nerve fiber layers of the retina, concentrated in the perimacular and perivascular areas (84). In a subset of 20 patients and 20 HC, hyperintense dots were detected with FAF following turmeric (Phytosome-Meriva) administration. Additional examination with OCT showed that the deposits were more prominent in MCI patients (84). In 2016, a study by Snyder et al. used OCT to explore whether retinal anatomic alterations are visible in pre-clinical stages of AD (48). A comparison between neocortical amyloid aggregation (florbetapir PET imaging) and various retinal sd-OCT markers of possible disease burden revealed that the surface area of retinal inclusion bodies significantly increased as a function of cortical amyloid burden (48).

Notably, Koronyo et al. (26) recently employed proprietary curcumin administration to fluorescently label amyloid deposits in the retina of 10 AD patients as compared to 2 patients with AMD and 6 HC (26). Imaging subjects with a modified SLO, prior to and after curcumin oral uptake, allowed for the detection of curcumin fluorescence signal and amyloid deposits with high resolution (Figure 1E). In this proof-of-concept clinical study, the feasibility to detect significant augmentation in retinal amyloid burden in a subset of AD patients compared to matched HC was demonstrated (Figure 1F) (26). Further, the team utilized focal scanning by OCT and revealed specified localization of amyloid deposits above retinal pigment epithelium and in the outer layers of the retina (Figures 1H,I). Importantly, this was in the absence of any maculopathy and was different than the picture in AMD patients (26). Consistent with histological examinations, retinal amyloid deposits measured *in vivo* were frequently concentrated in the mid- and far-periphery of the superior hemisphere (Figures 1E,G), an area that shows significant RNFL thinning in AD patients by OCT studies. While OCT has been presented as a feasible method of detecting neurodegeneration, these recent studies may implicate a new enhanced application of OCT to provide layer localization of the identified retinal plaques by SLO in MCI and AD patients.

## OCT in Other Neurodegenerative Disorders

### Parkinson’s Disease

As the second most common neurodegenerative disease, PD is characterized by motor dysfunctions such as tremor, rigidity, and bradykinesia in addition to psychological impairments including cognitive deficits, mood variability, and dementia (117–119). Like AD patients, individuals suffering from PD experience visual disturbances, which, in particular, manifest as hallucinations and reduced VA (16, 58, 85–87, 120, 121). Physiological injury primarily involves the loss of cerebral dopamine, a neurotransmitter involved in physical movement and reward-seeking behavior. Initial studies examining the retina of PD patients by OCT reported a significant reduction in RNFL thickness (inferotemporal, superotemporal, inferior, and temporal quadrants) (19, 76, 82, 87, 88, 90, 122), macular thickness (85, 89, 91), and foveal thickness (123) when compared to healthy subjects. OCT abnormalities in PD patients and their correlations with disease progression alongside visual dysfunction are summarized in Tables 1 and 2.

Notably, a large, longitudinal study evaluated the retina of PD patients *via* OCT and revealed that these patients exhibit a significant progressive thinning of the RNFL in the superotemporal and inferotemporal regions as well as a reduction in macular thickness against healthy controls (86). After a 5-year follow-up, the results indicating a significant reduction in the superotemporal and temporal regions were confirmed. Importantly, there was a moderate correlation between superotemporal thinning and visual dysfunction as well as disease severity (86). Although one study was unable to find differences between PD patients and matched controls in RNFL and GCL thickness and in macular volume (45), other studies supported these findings (19, 76, 82, 85–91, 122, 123). With the recent studies demonstrating a progressive retinal degeneration and the discovery of disease-specific α-synuclein in the retina of PD patients (29, 89–91, 120, 124–126), the use of retinal imaging in measuring PD-related neurodegeneration is promising, especially OCT. So far, OCT has seemingly provided an effective means of assessing structural changes in the retina in order to identify suitable detection pathways that may allow for earlier diagnosis, treatment, and slowing of disease progression.

### Huntington’s Disease

Patients with HD, a neurodegenerative disease with a largely genetic component, experience a wide array of symptoms including involuntary muscular movements known as chorea, dementia, and behavioral disturbances (119, 127, 128). In particular, the spreading of the disease follows an autosomal dominant inheritance with a genetic HTT mutation elongating the CAG repeat, with longer repeats translating to earlier onset [recently reviewed in Ref. (129)] (130–132). Kersten et al. (133) examined sd-OCT scans of the macula and pRNFL in 26 HD and 29 healthy patients (133). Markedly, though the research team found no difference in average pRNFL between HD and healthy individuals, they demonstrated a significant reduction in temporal pRNFL thickness in patients compared to healthy controls (62.3 vs. 69.8 mm; *p* = 0.005). In addition, the research team also noted that disease duration negatively correlates with both pRNFL thickness and macular volume, suggesting that increased length of HD affliction significantly thins the pRNFL and reduces macular size. In line with the latter correlation analysis of macular volume, Andrade et al. (134) evaluated macular and pRNFL thickness in patients with HD and normal control individuals by use of sd-OCT (134). Findings from this study suggested that HD patients express significantly decreased average (231.3 vs. 296.2; *p* = 0.033), central (341.8 vs. 252.0; *p* = 0.015), and inferior (225.3 vs. 313.8; *p* = 0.007) macular choroidal thickness in comparison to healthy controls (134). In summary, these studies suggest that noninvasive detection of HD signs in ocular tissues may provide a useful modality for diagnosis and following disease progression.

### Multiple Sclerosis (MS)

Multiple sclerosis, an autoimmune disease represented by demyelination of axons and disruptions to inflammatory homeostasis, has also been identified by neuronal death (135–137). Given that patients with MS exhibit inflamed eyes, past reports have suggested that cerebral pathology may mirror ocular manifestations, further enriching the value of using optical imaging mechanisms in diagnosing the disease. Notably, the ocular inflammation present in MS patients has recently been associated with RNFL thinning and neuronal degeneration (138). Similar to the results shown for AD, OCT imaging of MS patients has demonstrated a thinning of the RNFL compared to controls. Significant thinning of average and temporal RNFL thickness was found in MS-related optic neuritis patients (80, 93, 139) and in MS patients without optic neuritis (140, 141). A study by Fisher et al. (142) demonstrated that RNFL thickness is reduced significantly among MS patients (92 µm) vs. controls (105 µm) and is particularly reduced in diseased eyes with a history of optic neuritis (85 µm) (142). Studying a subgroup of MS patients with the macular thinning predominant phenotype showed conflicting results; while Saidha et al. (143, 144) reported that primary retinal pathology detectable by OCT defines this subset of patients with MS from controls (143, 144), a study by Brandt et al. (145) did not support this conclusion (145).

In addition, several earlier OCT studies have not only enhanced the understanding of ocular indicators for MS, but have also helped to elucidate the disease’s pathologically progressive timeline (141, 146–148). For instance, OCT examination by Henderson et al. (141) in patients with secondary progressive MS showed a more advanced disease stage compared to those with primary progressive MS. This study further indicated that RNFL thickness in the superior and temporal quadrants as well as macular volume exhibit considerable diminutions in secondary progressive MS patients (*p* = 0.045, *p* < 0.001, and *p* = 0.005, respectively) as compared to controls, but only in the temporal quadrant (*p* = 0.008) in patients with primary progressive MS, offering a means of distinguishing between the two disease stages (141). Accordingly, the use of OCT in MS promises to be highly constructive given not only the ability to detect retinal markers tied to the disease but also the variability in ocular pathologies based on disease advancement.

### OCT in Neurodegenerative Retinopathies

Similar to neurodegenerative diseases such as AD, PD, and MSON, open-angle glaucoma patients have been shown to experience significantly reduced RNFL thickness by use of OCT, with thinner RFNL corresponding to hindered visual performance as measured by PERG, an exam assessing retinal function (93). Specifically, Parisi et al. demonstrated that the PERG recordings from glaucoma, ocular hypertension, AD, and MSON patients exhibited smaller amplitudes and prolonged implicit times as compared to healthy individuals, suggesting that such diseases similarly impair visual function (93). Like AD patients, normal-tension glaucoma patients also experience this decrease in pRFNL thickness (*p* = 0.004) in addition to reduced macular ganglion cell complex (GCC) thickness (*p* = 0.006) and augmented global loss volume (*p* < 0.001) (71). Markedly, Eraslan et al. (71) further indicated the pertinence of considering retinopathy in AD by showing no significant difference between the pRNFL and GCC thickness of AD patients and normal-tension glaucoma patients *via* OCT imaging (*p* > 0.05).

Other ocular diseases that involve a neuronal degenerative component are AMD and diabetes retinopathy; a common feature of AMD is degeneration of retinal neurons (149, 150), and within diabetes are a variety of pathologies, which include targeted neuronal death (151). Regardless of the non-significant difference found in RFNL and GCL thickness between healthy individuals and type 1 diabetics, Carnevali et al. (152) sparked interest in examining diabetic retinopathy with OCT by divulging the ability to detect microvascular alterations that may appear before the manifestation of more prominent neuro-retinopathologies associated with diabetes. Specifically, diabetic patients exhibited reduced ocular vessel density in comparison to healthy controls (0.464 vs. 0.477, *p* = 0.005), further enhancing the relevance of OCT as an efficient imaging tool for early disease detection and preventative care (152). Additionally, in comparison to other imaging modalities such as blue-light and near-infrared FAF, OCT has been noted as the most correct and reproducible tool in examining impairments to the geographic atrophy of the fovea for AMD diagnosis (153). Furthermore, sd-OCT allows not only for investigation of structural changes in the retina, but also for variations occurring at the cellular level in order to accurately diagnose AMD (154). Overall, OCT analysis allowing for detection of diverse pathologies may, in conjunction with disease-specific biomarker imaging, facilitate differential diagnosis of various retino- and neurodegenerative diseases.

## Concluding Remarks

A revolutionary idea has arisen in recent years suggesting representation of brain pathology in retinal tissue, evidenced by similar manifestations such as hallmark depositions and neuronal cell death. The AD-specific misfolded protein, Aβ plaque, is thought to drive tauopathy and inflammation (11, 155), cocontributing to the vast synaptic and neuronal loss (14), and making Aβ the holy grail of AD diagnostic biomarkers. In the retina, the most extensively reported neural degeneration is of RGCs and the corresponding axonal thinning of the RNFL, as demonstrated mainly by OCT imaging. The apparent correlations between OCT-structural findings and visual and cognitive functions in AD patients support its utilization in assessing neurodegenerative incidence and progression. However, while the geometric distribution of progressive tissue atrophy is profound in the superior and inferior quadrants of AD retina, the potential overlap of the structural changes between various neurodegenerative diseases, such as PD, HD, and MS may instill an inherent limitation in differential diagnosis that should be addressed in future studies.

Recent estimates suggest that over 45 million individuals suffer from AD and associated dementia worldwide, and this number may more than double by 2040 (5, 6). This is a major concern for the aging population, as incidence rises sharply after 65 years of age, affecting roughly 50% of individuals aged 85 and older (5, 6). Despite considerable progress toward detection of AD biomarkers, practical diagnostic methods suitable for wide clinical deployment are greatly needed. Existing brain amyloid imaging technologies are the gold standard for AD diagnosis (156), but present challenges such as high costs, limited accessibility, and exposure to radioactive isotopes (13, 157, 158), and thus are currently unfit for large-scale population screening or monitoring response to therapies (13, 159–161). With the reported colocalization of retinal Aβ and ganglion cell degeneration, a combined method to detect hallmark Aβ deposits *via* fundus optical imaging with assessment of structural abnormalities by OCT may prove to be a superior approach for screening at-risk populations, assessing disease progression, and evaluating therapeutic efficacy.

## Author Contributions

JD, TT, YK, and MK-H: data collection and summary and discussion of intellectual content, manuscript writing, and editing. JD and MK-H: conceptual ideas, figure creation, and main writing. KB: discussion of intellectual content and editing. MK-H: supervision and final approval of manuscript.

## Conflict of Interest Statement

The authors declare that the research was conducted in the absence of any commercial or financial relationships that could be construed as a potential conflict of interest. The reviewer NB and handling editor declared their shared affiliation.

## Abbreviations

AMD, age-dependent macular degeneration; AD, Alzheimer’s disease; ADAS-cog, Alzheimer’s Disease Assessment Scale-cognition; Aβ, Amyloid beta-protein; CNS, central nervous system; CDR, Clinical Dementia Rating; CV, color vision; CSV, contrast-sensitive vision; fd-OCT, fourier/frequency domain OCT; FAF, fundus autofluorescence; GCL, ganglion cell layer; HD, Huntington’s disease; IPL, inner plexiform layer; MCI, mild cognitive impairment; MMSE, Mini-Mental State Examination; MS, multiple sclerosis; NFTs, neurofibrillary tangles; OCT, optical coherence tomography; PD, Parkinson’s disease; PERG, pattern electroretinogram; pRNFL, peripapillary retinal nerve fiber layer; RAI, retinal amyloid index; RGC, retinal ganglion cell; RNFL, retinal nerve fiber layer; RPE, retinal pigment epithelium; sd-OCT, spectral domain OCT; VA, visual acuity.
